# A machine learning-based procedure for leveraging clickstream data to investigate early predictability of failure on interactive tasks

**DOI:** 10.3758/s13428-022-01844-1

**Published:** 2022-06-01

**Authors:** Esther Ulitzsch, Vincent Ulitzsch, Qiwei He, Oliver Lüdtke

**Affiliations:** 1 IPN – Leibniz Institute for Science and Mathematics Education, Educational Measurement, Olshausenstraße 62, 24118 Kiel, Germany; 2 Technical University Berlin, Berlin, Germany; 3 Educational Testing Service, Princeton, NJ USA; 4 Center for International Student Assessment, Munich, Germany

**Keywords:** Interactive tasks, Early prediction, Extreme gradient boosting, Time-stamped action sequences, Clickstreams, PIAAC

## Abstract

Early detection of risk of failure on interactive tasks comes with great potential for better understanding how examinees differ in their initial behavior as well as for adaptively tailoring interactive tasks to examinees’ competence levels. Drawing on procedures originating in shopper intent prediction on e-commerce platforms, we introduce and showcase a machine learning-based procedure that leverages early-window clickstream data for systematically investigating early predictability of behavioral outcomes on interactive tasks. We derive features related to the occurrence, frequency, sequentiality, and timing of performed actions from early-window clickstreams and use extreme gradient boosting for classification. Multiple measures are suggested to evaluate the quality and utility of early predictions. The procedure is outlined by investigating early predictability of failure on two PIAAC 2012 Problem Solving in Technology Rich Environments (PSTRE) tasks. We investigated early windows of varying size in terms of time and in terms of actions. We achieved good prediction performance at stages where examinees had, on average, at least two thirds of their solution process ahead of them, and the vast majority of examinees who failed could potentially be detected to be at risk before completing the task. In-depth analyses revealed different features to be indicative of success and failure at different stages of the solution process, thereby highlighting the potential of the applied procedure for gaining a finer-grained understanding of the trajectories of behavioral patterns on interactive tasks.

## Introduction

Interactive tasks mirror dynamic, real-life environments, aiming at a more realistic assessment of what examinees know and can do. Prominent examples for these environments are the simulated email, web pages, and spreadsheet environments employed in the Programme for the International Assessment of Adult Competencies (PIAAC; OECD, 2013) to measure problem solving in technology-rich environments (PSTRE), or the interactive problem-solving tasks administered in the Programme for International Student Assessment 2012 (PISA; OECD, 2014). Being computer-administered, assessments using interactive tasks support logging clickstream data in the form of time-stamped action sequences, documenting the type, order, and timing of the actions examinees executed when trying to solve the given tasks. This rich source of additional data comes with great potential for a nuanced understanding of response processes, and allows to move from investigating *whether* to *how* examinees solved a task (Greiff, Wüstenberg, & Avvisati, 2015), for instance, by identifying typical strategies (e.g. He, Borgonovi, & Paccagnella, 2021; Ulitzsch et al.,, 2021b; Vista, Care, & Awwal, 2017; Wang, Tang, Liu, & Ying, 2020; Zhu, Shu, & von Davier, 2016) or investigating which behavioral patterns distinguish success from failure on a task (e.g. Han, He, & von Davier, 2019; He & von Davier, 2015; Qiao & Jiao, 2018; Salles, Dos Santos, & Keskpaik, 2020).

In this study, we introduce a procedure for systematically investigating whether and how early performed actions as well as the time required for their execution already contain sufficient information for predicting the outcome of examinees’ behavioral trajectories, that is, success or failure, and for identifying examinees at risk of failure before they complete the task. To this end, we make use of early-window clickstream data, i.e., time-stamped action sequences comprising only initially performed actions and the associated time stamps. We consider predictions to be useful if accurate predictions can be achieved at stages where the majority of examinees have the greater part of their solution process still ahead of them and the majority of examinees who failed could potentially be detected to be at risk before completing the task. Investigating early predictability comes with great potential for a finer-grained understanding of how examinees approach interactive tasks and may potentially aid in improving the testing procedure. More specifically, first, investigating early-window clickstream data may improve our understanding of behavioral patterns of early interactions with interactive tasks (e.g., initial exploration or planning behavior) that distinguish behavioral trajectories of examinees succeeding or failing on a task. This knowledge can then be used to refine theories on test-taking behavior or be employed in interventions that aid students in improving their skills for initial exploration of complex problem-solving tasks. Second, such analyses support investigating whether it is possible to dynamically track examinees’ risk of failure as they interact with the task. Once risk of failure can reliably be inferred from early interactions, this knowledge may—when combined with a good understanding of the sources of failure—be put into action by providing early support in real time such as hints or reformulations of the task that may aid examinees at risk of failing to successfully complete the task.

Although rarely encountered in the context of interactive tasks, the objective of predicting behavioral outcomes from early-window clickstream data is not unknown in the behavioral sciences and has been successfully addressed in various applications, ranging from predicting grades or dropout from early uses of online learning management systems (e.g. Baker, Lindrum, Lindrum, & Perkowski, 2015; Lykourentzou, Giannoukos, Nikolopoulos, Mpardis, & Loumos, 2009; Mongkhonvanit, Kanopka, & Lang, 2019; Whitehill, Williams, Lopez, Coleman, & Reich, 2015) to predicting purchase events from early browsing behavior on e-commerce platforms (e.g. Awalkar, Ahmed, & Nevrekar, 2016; Hatt & Feuerriegel, 2020; Requena, Cassani, Tagliabue, Greco, & Lacasa, 2020; Toth, Tan, Di Fabbrizio, & Datta, 2017). In the present study, we build on these previously applied procedures for early-window clickstream data and explore whether and how they can be adapted to the context of early prediction of behavioral outcomes on interactive tasks in general and failure in particular.

In what follows, we first review previous research on using process data to better understand behavioral patterns differentiating correct from incorrect responses. Subsequently, we provide a short overview on approaches to early prediction of shopper intent on e-commerce websites. We then use these approaches as a blueprint and starting point for introducing a procedure for systematically investigating early predictability of behavioral outcomes on interactive tasks. The procedure is outlined by assessing early predictability of failure on two tasks from the PIAAC PSTRE domain. Finally, we discuss implications and identify potentials for future work.

### Using clickstream data to differentiate correct from incorrect responses

Posing a rich description of how examinees attempted the administered tasks, clickstream data from computer-based interactive tasks have recently gained much attention in psychometrics, psychology, and educational sciences. Within this stream of research, both theory-driven and exploratory approaches to investigating behavioral patterns related to success and failure on interactive tasks emerged. Herein, however, the predominant aim has been to investigate behavioral patterns rather than to predict behavioral outcomes.

#### Theory-driven approaches

Theory-driven approaches commonly aim at corroborating theories on solution and test-taking behavior. Based on subject-matter theory, clickstream data are used for the construction of behavioral indicators. Examples for such indicators are the application of specific strategies (such as vary-one-thing-at-a-time, VOTAT; Greiff et al.,, 2015; or other expert-defined strategies as in Hao, Shu, & von Davier, 2015; He, Borgonovi, & Paccagnella, 2019), the degree of automation of procedural knowledge as indicated by the time spent on automatable subtasks (e.g., drag-and-drop events; Stelter, Goldhammer, Naumann, & Rölke, 2015), planning behavior as indicated, e. g., by the time required for performing the first action (Albert & Steinberg, 2011; Eichmann, Goldhammer, Greiff, Pucite, & Naumann, 2019), or disengaged behavior as indicated by short times spent on task and few actions (Sahin & Colvin, 2020). Subsequently, these behavioral indicators can be related to performance in order to investigate whether the considered behaviors are related to successful task completion as hypothesized.

Applications of theory-driven approaches have markedly deepened the understanding and refined theories of test-taking behavior on interactive tasks. For predicting the outcomes of behavioral trajectories, however, purely theory-driven approaches are limited. First, for the construction of theory-derived indicators, clickstreams are scanned for occurrences of specific strategies. Hence, when prediction rather than corroborating theories is the primary research objective, potentially useful information is discarded. Second, some of these indicators may be constructed only on the basis of longer sequences and/or when the solution process is already at more advanced stages, such that the behavioral patterns used for indicator construction may not often be encountered in early-window clickstream data. VOTAT, for instance, is a complex strategy that manifests itself in sequences of actions that may occur only in later stages of the solution process when examinees have acquainted themselves with the task environment.

#### Exploratory approaches

In recent years, a plethora of exploratory approaches to identifying features distinguishing correct from incorrect clickstreams has been developed and applied (Chen, Li, Liu, & Ying, 2019; Han et al.,, 2019; He & von Davier, 2016; Qiao & Jiao, 2018; Salles et al.,, 2020; Ulitzsch, He, & Pohl, 2021a). Features derived from clickstreams comprise generic features commonly used in sequence mining or natural language processing (e.g., *n*-grams as in He & von Davier, 2015, 2016; Liao, He, & Jiao, 2019; Ulitzsch et al.,, 2021a), task-specific features, created based on subject-matter knowledge on behavioral patterns to be expected on the task (Chen et al., 2019; Salles et al., 2020), or a combination of the two (Qiao & Jiao, 2018; Han et al., 2019). These features are then fed to classifiers or prediction models, or analyzed using sequence mining techniques to identify features that best distinguish correct from incorrect clickstreams.

Note that commonly the objective of such approaches is not prediction but rather to better understand examinees’ attempts to solve the administered tasks by uncovering key behavioral patterns that distinguish success from failure. Aimed at gaining insights on the whole solution process, these approaches leverage the whole of information contained in collected clickstreams—from opening the task to proceeding to the next one. As the actions performed on interactive tasks are an inherent part of the solution process, correct and incorrect clickstreams have been found to be well distinguishable. For a PISA 2012 problem-solving task, for instance, Qiao and Jiao (2018) reported specificity and sensitivity of more than .90 for various classifiers being fed *n*-grams extracted from action sequences. Analyzing an interactive math item from the French Cycle des Évaluations Disciplinaires Réalisés sur Échantillons (subject-related sample-based assessment cycle; CEDRE), Salles et al., (2020) obtained an area under the receiver operating characteristic curve (AUC ROC) value of .78 from random forest analyses using theory-derived, task-specific features. Such good performance, however, may not necessarily be achievable for predictions based on early-window clickstream data, which are the focus of the present study. First, behavioral patterns distinguishing success from failure may be encountered only at later stages of the solution process, while differences in the very first actions, stemming, for instance, from initial exploration behavior, may be less pronounced. Second, information contained in early-window clickstream data from interactive tasks may be rather sparse. For instance, across the 14 tasks of the PIAAC 2012 domain, average sequence length ranged from 10.8 to 96.9 (Tang, Wang, Liu, & Ying, 2020b). If we were to predict outcomes of behavioral trajectories after what would, on average, be the middle of the solution process, on some tasks, predictions would need to be made on the basis of as few as five actions and the associated timing information.

#### Predictive approaches

So far, the predominant goal of analyses of clickstream data has been to gain a better understanding of behavioral patterns rather than making predictions. Nevertheless, just recently, predictive approaches started to emerge.

Tang, Wang, He, Liu, and Ying (2020a) investigated whether action sequence data from one PIAAC PSTRE task can predict performance on another one. To that end, the authors determined the discrepancy between action sequences from each PIAAC PSTRE task by drawing on a dissimilarity measure originating from clickstream analysis and subsequently extracted item-specific latent features via multidimensional scaling. Using logistic regression, the authors then investigated whether features derived from one task can predict success or failure on another one over and above performance on the predicting task. For most of the item pairs, Tang et al., (2020a) reported a marked improvement in prediction accuracy when features were included, highlighting the vast potential of information contained in sequence data for predicting the performance of examinees.

Chen et al., (2019) proposed a model-based approach for dynamic prediction of behavioral outcomes. The authors proposed to include features as time-varying covariates in an event history model, which at any given time of the solution process can be used to predict outcomes of the solution process, i.e., success or failure as well as time spent on the task. Their study is an important contribution as it showcased and initiated the discussion on the utility of clickstream data for dynamic predictions of behavioral outcomes on interactive tasks. Nevertheless, Chen et al., (2019) critically remarked that although employing a prediction model rather than using black-box machine learning methods allows retrieving interpretable parameters, it comes at the price of strong assumptions on data-generating processes which, given the complexity of clickstream data, renders the model likely to “not most closely approximate the data-generating process” (Chen et al.,, 2019, p. 4), potentially yielding biased predictions. Among others, these assumptions concern the functional form of the relationship between considered features and behavioral outcomes. Further, regression weights are assumed to be time-invariant, implying that the considered features are equally predictive at different stages of the solution process. This must not necessarily be the case. Actions related to task exploration, for instance, may be positively related to success at early stages, capturing examinees’ willingness to thoroughly explore the task environment, but may be indicative for risk of failure at later stages of the solution process, when such actions are no longer beneficial for successful task completion. Analyzing a PISA 2012 problem-solving task, Chen et al., (2019) retrieved a satisfactory AUC ROC value of .72 only at later stages of the solution process when the median time spent on the task had already passed, which may be considered as a benchmark for subsequent studies.

### Using early-window clickstream data for shopper intent prediction

In fields where clickstream data is a more established source of behavioral data, predicting behavioral outcomes from early-window clickstream data is a common problem statement. In the present study, we turn our attention to procedures employed in the context of predicting behavioral outcomes based on clickstream data from e-commerce websites. In this vein of research, clickstream data is commonly used for predicting whether users are at risk for leaving the page without purchases (see Awalkar et al.,, 2016; Bertsimas, Mersereau, & Patel, 2003; Hatt and Feuerriegel, 2020; Requena et al.,, 2020; Toth et al.,, 2017, for examples). Early detection of such risks may trigger automated interventions, such as offering discounts that may nudge customers into purchasing. To that end, a plethora of supervised classifiers has been employed, ranging from predictive models for sequential data such as hidden Markov models (as in Hatt & Feuerriegel, 2020) or recurrent neural networks (as in Toth et al.,, 2017) to classifiers trained on features derived from clickstream data such as extreme gradient boosting or support vector machines (as in Requena et al.,, 2020). Features considered comprise information on the action level such as uni- and bigrams (Requena et al., 2020), aggregates such as the number of performed clicks or the maximum time elapsed between subsequent clicks as well as metadata such as the day of the week when the session was initiated (Awalkar et al., 2016). Research on predictions of behavioral outcomes has repeatedly demonstrated that clickstream data is well suited for making accurate predictions at relatively early points in time based on rather sparse data.

Data structures from e-commerce websites can be expected to resemble those encountered in interactive tasks, rendering it worthwhile to investigate whether procedures applied in the context of e-commerce also perform well in the context of interactive tasks. First, interactive tasks such as those employed in the PIAAC PSTRE domain oftentimes mirror interfaces of web applications to evoke real-life problem-solving behavior. Second, clickstreams from e-commerce websites tend to be rather short. Requena et al., (2020), for instance, based their analyses of shopper intent prediction on browsing sessions with action sequences of length 5 to 155, closely resembling typical ranges encountered in clickstream data from interactive tasks. Across all 14 tasks of the PIAAC PSTRE domain, for instance, the minimum action sequence length was 3 and maximum action sequence length ranged from 51 to 398 (Zhang, Tang, He, Liu, & Ying, 2021).

Due to these resemblances in typical data structures, procedures employed for investigating the early predictability of shopper intent pose a promising tool for investigating the early predictability of failure or success on interactive tasks. In the present study, we draw on and adapt procedures that have recently been employed by Requena et al., (2020) in their systematic and exhaustive study of early shopper intent prediction. Requena et al., (2020) created multiple subsets of action sequences that were trimmed to all but those actions that fell into a given early window. Next, the authors compared the performance of multiple machine learning algorithms on these subsets to investigate at which point early-window action sequences contained sufficient information to achieve accurate predictions. Among others, Requena et al., (2020) achieved good results with extreme gradient boosting, where AUC ROC values exceeded .70 as soon as early action sequences were of at least length seven.

## Objective and research questions

Adapting machine learning-based procedures originally employed by Requena et al., (2020) for investigating early predictability of shopper intent on e-commerce websites, the present study introduces and showcases a procedure for the systematic investigation of early predictability of behavioral outcomes on interactive tasks in educational assessment. When introducing the procedure, we suggest features that may be derived from clickstream data from interactive tasks as well as measures to be tracked that aid in evaluating the quality and utility of early predictions. We outline the procedure by investigating the potential of early-window clickstream data for early prediction of risk of failure on two PSTRE tasks from PIAAC 2012, addressing the following research questions:

1. Establishing a baseline: How well can customary supervised classifiers on the basis of features constructed from complete clickstream data, capturing the whole solution process, identify failure on the task?
2. Investigating the accuracy of early predictions: How early in terms of a) the number of performed actions as well as b) elapsed time can customary supervised classifiers on the basis of features constructed from early-window clickstream data accurately predict failure on the task?
3. Investigating feature importance: Which features constructed from early-window clickstream data display the highest predictive importance at different phases of the solution process?

## Materials and methods

### Data

We made use of clickstream data from the items U23 (“Lamp Return”) and U02 (“Meeting Rooms”) from the PIAAC 2012 PSTRE domain. In PIAAC 2012, problem-solving items were administered with fixed positions and without time limits. “Meeting Rooms” is located in the middle of the second problem-solving cluster (PS2), while “Lamp Return” is administered at the very end of PS2. Hence, when approaching “Meeting Rooms” and “Lamp Return”, examinees were already exposed to different PIAAC PSTRE task environments and had the opportunity to accumulate pre-familarity with these environments. We chose these items as they strongly differ in their difficulty as well as in the amount of initial task exploration required prior to performing key actions for solving the task, both potentially impacting early predictability. Very difficult or very easy items yield highly imbalanced data sets which may challenge classifiers (see, e.g., Ruisen et al.,, 2018). The amount of initial task exploration required prior to performing key actions may impact how distinguishable early-window clickstream data associated with success or failure are because differences in initial exploration behavior may be less pronounced and differences in performing key actions for solving the task may emerge only at later stages of the solution process.

**Fig. 1:**
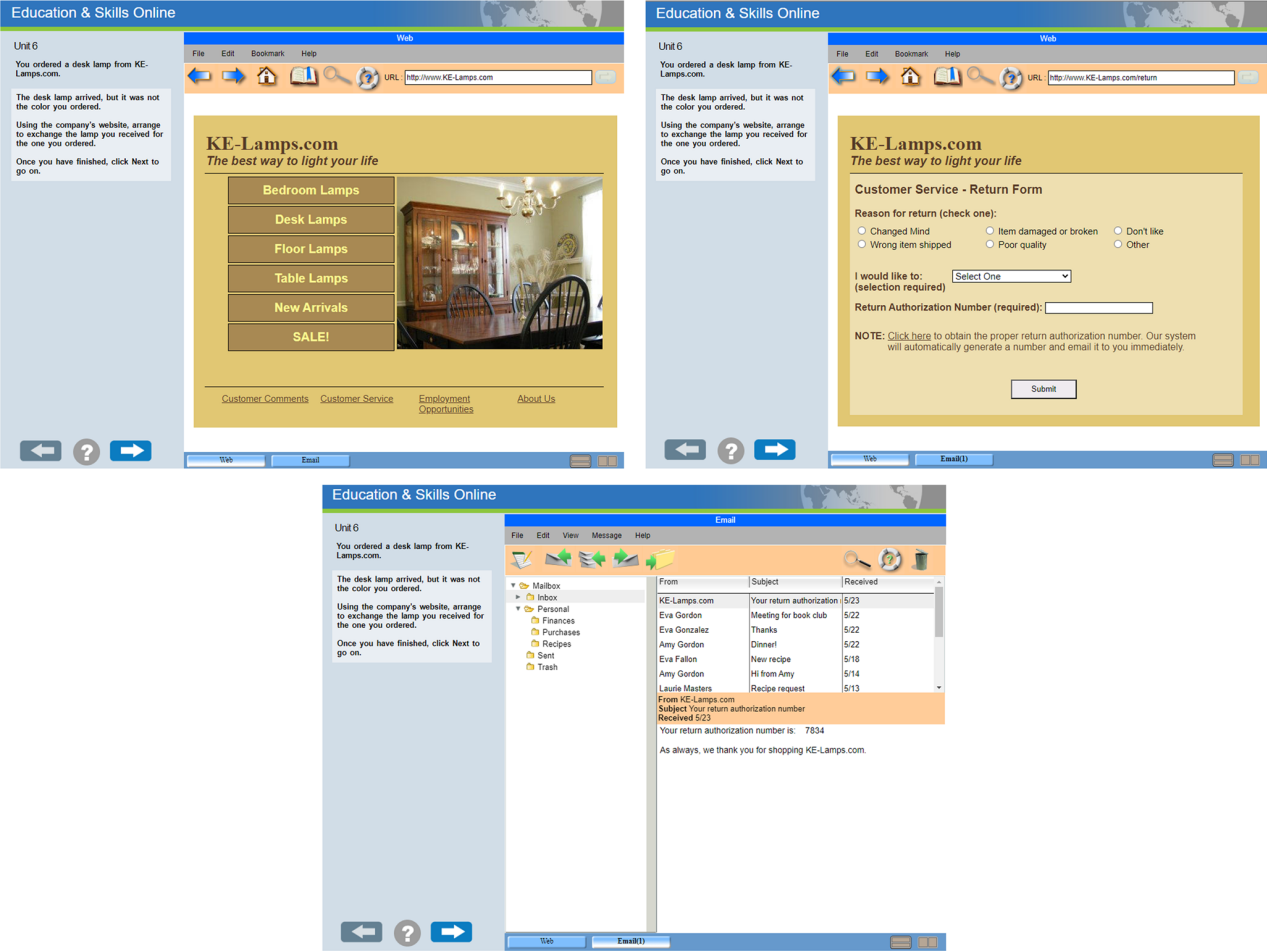
An example item with the theme of lamp return from the Education and Skills Online Assessment, which shares the similar item interface structure with the PIAAC PSTRE tasks. Source: https://www.oecd.org/skills/ESonline-assessment/takethetest/#d.en.367010

“Lamp Return” involves both web page and email environments and requires examinees to navigate through an online lamp shop to complete an explicitly specified consumer transaction. To that end, examinees have to submit a request, retrieve an email message, and fill out an online form. Examinees receive partial credit if at least one of the fields of the online form is filled out correctly. Figure 1 displays an example item with email and web environments (from the Education and Skills Online Assessment) that shares a comparable item interface with the PIAAC item “Lamp Return”. “Meeting Rooms” involves email, web, and word processor environments^1^ and requires examinees to navigate through emails, identify relevant requests for meeting room reservations, and subsequently submit these meeting room requests via a simulated online reservation site. A conflict between one request and the existing schedule presents an impasse to be resolved.

“Lamp Return” and “Meeting Rooms” are located at Proficiency Levels 2 and 3, respectively,^2^ and, with item difficulties of 321 and 346, respectively, pose items of medium and high difficulty (OECD, 2013). For getting to and filling out the lamp return form, it is not necessary to exhaustively explore the task’s environment. As such, the item can be solved in a rather linear manner and only requires a minimum of 17 actions (including actions performed for filling out the return form) for receiving full credit (He et al., 2021). Key actions required for successful task completion can therefore be expected to be commonly encountered in early-window clickstream data associated with successful task completion. This is different for “Meeting Rooms”, which requires examinees to seek and integrate information from multiple environments before filling out the meeting room reservation forms. Due to the higher necessity of initial task environment exploration, “Meeting Rooms” requires a minimum of 25 actions for receiving full credit (He et al., 2021). Initial task exploration is likely to be non-linear, with examinees switching between different environments to compare and integrate the displayed information.^3^ Key actions required for successfully submitting the reservation forms may therefore be commonly encountered only at later stages of the solution process. Based on these consideration, we expected early predictability for “Lamp Return” to be less challenging than for “Meeting Rooms”.

We analyzed clickstream data from examinees from Ireland, Japan, the Netherlands, the United Kingdom, and the United States who were administered “Lamp Return” and/or “Meeting Rooms” of the PIAAC 2012 PSTRE domain. Data from 761 and 920 examinees who proceeded to the next task without performing any actions on “Lamp Return” and “Meeting Rooms”, respectively, were excluded. A total of 6,791 (“Lamp Return”) and 6,629 (“Meeting Rooms”) clickstreams were considered for further analyses. As the objective of this study was to identify examinees at the risk of failure, lacking the understanding, skills, and/or motivation to fulfill at least some of the task’s requirements, we scored partially correct as correct. On “Lamp Return”, 3,134 of examinees who performed at least one action failed and 3,657 (partially) succeeded. On “Meeting Rooms”, 3,957 examinees failed and 2,672 (partially) succeeded.

Examinees who failed in solving either “Lamp Return” or “Meeting Rooms” spent less time on the tasks and performed fewer actions than examinees who (partially) succeeded. Excluding the action “Start” as well as the final bigram 〈 “Next”, “NextOK” 〉 (proceeding to the next task and confirming this action), the median and middle 50% range of action sequence length on “Lamp Return” was 11 [7; 19] when associated with failure and 30 [24; 41] when associated with (partial) success. Examinees who failed on “Lamp Return” spent a median of 74 s with a middle 50% range of [48; 124] on the task, while examinees who (partially) succeeded required a median of 134 s with a middle 50% range of [102; 187] for doing so. The median and middle 50% range of action sequence length associated with failure and success on “Meeting Rooms” were 20 [9; 44] and 84 [66; 107], respectively. Median and middle 50% range of time spent on “Meeting Rooms” associated with failure and success were 117 [65; 218] and 394 [309; 518] s, respectively.

### Preprocessing

Actions that are not essential for successfully solving the task were recoded into aggregate-level categories (e.g., “write email”, “explore shop’s products”, or “open folders”). Further, we aggregated all actions that could be performed using different tools of the simulated environment but yielded the same result (e.g., “switch between email and web environment”, “submit request”). In total, this resulted in 27 categories of performable actions for “Lamp Return” and 31 categories for “Meeting Rooms”. Overviews of performable actions, relative frequencies of action sequences associated with success and failure containing the actions (sequence frequencies), absolute frequencies of actions within each response group (action frequencies), and median time to first occurrence are given in Tables 1 and 2. Note that details related to filling out the tasks’ online forms were stripped from the action sequences as from this information the score can directly be inferred, rendering prediction a trivial endeavor. On “Lamp Return”, for instance, we kept information that examinees selected a reason for their return request, but stripped information on which specific reason they selected. Likewise, on “Meeting Rooms”, we kept information that examinees selected a start time on the reservation form but eliminated information on the specific date and time. Further, we eliminated actions related to submitting forms and requests since these correspond to the submission of examinees’ solution prior to proceeding to the next task.

**Table 1:** Description, frequencies, and median time to first occurrence of performable actions on Item U23 (“Lamp Return”) by response group

Name	Description	Sequence frequency		Action frequency		Time to first occurrence	
		Success	Failure	Success	Failure	Success	Failure
GoToCustomerService	Go to customer service	1.00	0.67	4818	2435	26	30
GoToReturnForm	Go to return form	1.00	0.03	4296	125	68	50
EmailWeb	Switch between email and web environment	0.92	0.53	14608	5385	54	44
BackForward	Going back or forward	0.93	0.42	10315	3588	58	45
Reason	State reason for returning lamp on return form	0.99	0.00	3743	8	84	91
Exchange	State return modality on return form	1.00	0.00	3682	8	87	97
AuthBox	Fill in authorization number on return form	0.93	0.00	25621	46	107	110
Submit	Submit return form	0.93	0.00	4629	13	124	141
ObtainAuthNumber	Request authorization number	0.94	0.56	13219	6015	67	46
ViewAuthMail	Open email with authorization number	0.86	0.20	3771	912	92	75
Products	Explore shop’s products	0.40	0.58	3811	5491	26	22
FolderView	Open folder in email environment	0.35	0.15	7176	2805	70	79
CustomerServiceInfo	Explore information on customer service site	0.24	0.14	1348	651	76	74
MailView	Open email	0.22	0.13	9510	3396	63	65
SiteNotNeeded	Pop-up window “Content not needed for task”	0.20	0.38	1873	2977	54	40
CompanyInfo	Explore company information	0.18	0.15	907	686	59	56
Keystroke	Perform keystroke	0.16	0.10	5378	31933	92	119
Toolbar	Use toolbar	0.11	0.13	779	854	92	83
Menu	Use menu	0.10	0.07	725	402	102	94
CopyPaste	Copy and paste	0.06	0.01	611	104	99	117
NextCancel	Cancel proceeding to next item	0.03	0.04	134	121	126	76
WriteMail	Write email	0.02	0.10	618	2618	143	117
MoveEmail	Move email	0.02	0.02	132	110	102	93
Bookmark	Set bookmark	0.02	0.04	168	404	111	94
Help	Seek help	0.02	0.04	93	160	124	95
Search	Use search function	0.01	0.00	223	46	137	105
Sort	Use sort function	0.00	0.00	18	14	171	144

Sequence frequency: proportion of sequences within the response group containing the action at least once; action frequency: absolute frequency of action occurrences within the response group; time to first occurrence: median time to first occurrence in seconds within the response group

**Table 2:** Description, frequencies, and median time to first occurrence of performable actions on Item U02 (“Meeting Rooms”) by response group

Name	Description	Sequence frequency		Action frequency		Time to first occurrence	
		Success	Failure	Success	Failure	Success	Failure
MailView	Open email	1.00	0.88	31403	20940	33	32
Folder view	Open folder in email environment	0.70	0.79	11979	22400	41	38
EmailWeb	Switch between email and web environment	1.00	0.49	44437	13624	58	64
WordProcessor	Interact with word processor	0.81	0.35	8818	4631	73	74
Submit	Submit request	1.00	0.22	11530	3136	228	183
GoToReservation	Go to reservation site’s reservation form	1.00	0.27	37710	10902	126	105
GoToCalender	Go to reservation site’s calender	0.94	0.23	22595	4955	99	97
GoToMeetingRoom	Go to reservation site’s meeting room information	0.81	0.20	12192	3314	137	118
GoToUnfilled	Go to reservation site’s unfilled request notice form	0.69	0.09	8467	1189	280	197
GoToHome	Go to reservation site’s home	0.10	0.05	758	641	132	128
Dept	Select department on reservation form	1.00	0.22	8683	1815	211	169
Room	Select room on reservation form	1.00	0.22	7968	2644	195	151
StartTime	Select start time on reservation form	1.00	0.22	6500	1672	195	150
EndTime	Select end time on reservation form	1.00	0.22	6482	1663	199	155
ChangeDept	Change department on reservation form	0.04	0.01	113	23	354	346
ChangeRoom	Change room on reservation form	0.16	0.01	471	44	268	274
ChangeStartTime	Change start time on reservation form	0.03	0.00	81	27	355	321
ChangeEndTime	Change end time on reservation form	0.03	0.00	94	25	358	325
CancelChanging	Cancel changes on reservation form	0.07	0.00	215	22	298	371
Toolbar	Use toolbar	0.28	0.19	2356	2217	114	100
BackForward	Going back or forward	0.23	0.08	2020	1137	162	137
Menu	Use menu	0.16	0.14	892	1214	148	99
MoveMail	Move email	0.16	0.29	1939	9329	123	66
NewFolder	Create new folder	0.14	0.02	987	371	312	181
Keystroke	Perform keystroke	0.09	0.06	24491	21776	158	122
Help	Seek help	0.07	0.06	318	396	167	135
NextCancel	Cancel proceeding to next item	0.05	0.03	135	110	329	119
CopyPaste	Copy and paste	0.03	0.02	167	165	196	146
Bookmark	Set bookmark	0.01	0.01	60	65	161	180
Sort	Use sort function	0.01	0.01	62	134	85	96
Search	Use search function	0.01	0.01	79	153	176	123

Sequence frequency: proportion of sequences within the response group containing the action at least once; action frequency: absolute frequency of action occurrences within the response group; time to first occurrence: median time to first occurrence in seconds within the response group

### Creating early-window subsets

For investigating early prediction of failure, we considered early windows in terms of the number of performed actions *W*^*a*^ = {1,2,...,7} and elapsed time in seconds *W*^*t*^ = {20,30,40,50} for “Lamp Return”, and *W*^*a*^ = {1,2,...,9} and *W*^*t*^ = {20,30,...,70} for “Meeting Rooms”.^4^ Herein, the longest early windows considered (i.e., 7, respectively 9 actions, and 50, respectively 70 s) roughly correspond to the first quartile of action sequence length and time spent on task associated with failure, with the rationale being that within these early windows, the vast majority of examinees who failed could still be detected to be at risk before completing the task. With *a*_*i*_ and *t*_*i*_ giving examinee *i*’s action sequence length and time spent on task, for each early window, we created a subset containing only sequences of length *a*_*i*_ > *w*^*a*^, respectively associated with *t*_*i*_ > *w*^*t*^, and trimmed clickstreams comprising the subset to the first *w*^*a*^ actions and the associated timing information, respectively to those actions and the associated timing information performed within the first *w*^*t*^ seconds. For instance, the *w*^*a*^ = 4 data set contained only clickstreams of examinees who performed a total of at least five actions, and the first four actions and associated time stamps were employed for prediction. In analogy, the *w*^*t*^ = 30 data set contained only clickstreams associated with a time spent on task of more than 30 s, and only those actions and associated time stamps were employed for prediction that were performed within the first 30 s. Each of the resulting 7 + 4 + 9 + 6 = 26 data sets was used to evaluate the predictability of failure based on the information contained in the trimmed clickstreams. This procedure is adapted from Requena et al., (2020) and supports a systematic investigation of how early behavioral outcomes (i.e., failure in the present application) can accurately be predicted. Note that this procedure creates subsets that not only differ in the richness of features used for early prediction but also in the set of behavioral trajectories of interest, as only those clickstreams exceeding the early windows with respect to action sequence length, respectively time spent on task, are subject to prediction. As different subsets of behavioral trajectories are investigated for each early-window data set, predictability must not necessarily increase with an increasing early-window size and increasing richness of features.

### Feature extraction

We derived multiple generic features from clickstreams, related to the occurrence, frequency, and sequentiality of performed actions, and enriched these with features derived from the time elapsed until the execution of specific actions.

#### Time to first action

In the literature on problem solving, time to first action has often been discussed as an indicator of planning time and shown to be associated with successful task completion (Albert and Steinberg, 2011; Eichmann et al., 2019). We therefore included time to first action as a time-related feature derived from subject-matter theory on problem-solving behavior.

#### Action term-frequency–inverse-document-frequency weight

Occurrences of performable actions were represented as term-frequency–inverse-document-frequency (tf-idf) weights, a common measure employed in natural language processing (Salton, 1975; see He & von Davier, 2015, 2016; Ulitzsch et al.,, 2021a, for applications in the context of interactive tasks). Herein, the tf-idf_*gi*_ weight for action *g* occurring in sequence *i* is determined as follows

1 $$ \text{tf-idf}_{gi} = \begin{cases} [1+\log(\text{tf}_{gi})]\log(N_{w}/\text{df}_{g}) & \text{if } \text{tf}_{gi}\geq1\\ 0 & \text{if } \text{tf}_{gi}=0 \end{cases}, $$

where df_*g*_ gives action *g*’s document frequency (which in the given context corresponds to the number of sequences *g* occurs in), tf_*gi*_ gives the term frequency (i.e., the number of occurrences) of action *g* in sequence *i*, and *N*_*w*_ is the number of sequences in the respective early-window data set. The weight upweighs actions occurring in only few sequences and being associated with lower df_*g*_, while dampening the multiple occurrence of actions within the same sequence (i.e., those actions within sequence *i* having a high tf_*gi*_). For illustration, let us assume that within a given early window, Examinee 1 performed the action “EmailWeb” (switching between the email and web environment) tf_EmailWeb1_ = 3 times. Let us further assume that there are *N*_*w*_ = 500 clickstreams in the early-window data set, out of which df_EmailWeb_ = 300 contain the action at least once. This results in a tf-idf weight of $[1+\log (3)] \log (500/300) = 1.07$ for the action “EmailWeb” in Examinee 1’s sequence. If fewer examinees would have performed “EmailWeb”, say df_EmailWeb_ = 100, this would result in a higher tf-idf weight of 3.38.

#### Time to action’s first occurrence

For each performed action, we considered the time elapsed until its first occurrence (see Tables 1 and 2 for overviews). In the case that an action was not encountered in a given sequence, the time to its first occurrence was coded as missing. The inclusion of this feature follows the rationale that the time at which a given action is executed may be indicative of whether behavioral trajectories result in success or failure. For instance, at the beginning of the solution process, actions related to exploring the task environment may be beneficial, indicating examinees’ willingness and ability to get acquainted with the task’s requirements. At later stages of the solution process, however, such behavior may be an indicator of inefficient and unsystematic solution behavior and thus of risk of failure.

#### Bi- and trigrams

To take the sequentiality of early actions into account, we considered bi- and trigrams, i.e., contiguous subsequences of size two and three, respectively. For these, we used a simple one-hot enconding (i.e., the feature takes the value 1 if the bi-, respectively trigram, is contained in the early-window sequence and is set to 0 otherwise). We did not consider the frequency of occurrence of bi- and trigrams because—due to the few number of actions performed in early windows—bi- and trigrams were rarely encountered more than once in a given early-window sequence. We did not include one-hot encoded unigrams, as information on their occurrence is already contained in the action tf-idf weights as well as in the times to the action’s first occurrence.

#### Activity

For early-window data sets, we considered the time elapsed within the given early window when trimming by the number of performed actions and the number of performed actions within the given early window when trimming by elapsed time. These features can be seen as indicators of the intensity of examinees’ interactions with the task environment within the given early window.

### Monitored descriptives

For each early-window data set, we monitored several descriptives, aimed at describing a) how early the prediction is performed, and b) the utility of early prediction in terms of its capability to identify examinees to be at risk of failure before they complete the task. Further, we tracked the proportion correct in each early-window data set to gauge the degree of imbalancedness of the classification problem.

#### Earliness

We evaluated how early predictions can be performed based on a given early window—either defined in terms of the number of performed actions *w*^*a*^ or in terms of elapsed time *w*^*t*^—by using and adapting the Earliness metric employed by Requena et al., (2020). For any examinee with *a*_*i*_ > *w*^*a*^ and *t*_*i*_ > *w*^*t*^, Earliness metrics in terms of the number of performed actions and the elapsed time are given by

2 $$ \text{Earliness}^{a(a)}_{i} = 1-\frac{w^{a}}{a_{i}} $$

and

3 $$ \text{Earliness}^{t(a)}_{i} = 1-\frac{t_{i}^{w^{a}}}{t_{i}}, $$

respectively, when trimming with respect to actions. The equations change to

4 $$ \text{Earliness}^{a(t)}_{i} =1-\frac{a_{i}^{w^{t}}}{a_{i}} $$

and

5 $$ \text{Earliness}^{t(t)}_{i} =1-\frac{w^{t}}{t_{i}}, $$

respectively, when trimming with respect to time. Here, $t_{i}^{w^{a}}$ gives the time examinee *i* required for performing the first *w*^*a*^ actions and $a_{i}^{w^{t}}$ gives the length of examinees *i*’s action sequence up to time *w*^*t*^. The Earliness metrics provide the relative distance between the point at which the prediction is performed to the end of the sequence. For instance, for an early window in terms of the number of performed actions of *w*^*a*^ = 3, sequences of length *a*_*i*_ = 4 have $\text {Earliness}^{a(a)}_{i}=.25$, while sequences of length *a*_*i*_ = 30 have $\text {Earliness}^{a(a)}_{i}=.90$. For each early window, we tracked the Earliness metrics’ medians and middle 50% ranges.

#### Utility for risk detection

As a measure of the utility of risk detection at a given early window, we tracked the ratio of the number of incorrect clickstreams with *a*_*i*_ > *w*^*a*^, respectively associated with *t*_*i*_ > *w*^*t*^, to the number of incorrect clickstreams in the complete data set. This ratio corresponds to the proportion of examinees who failed that could be identified to be at risk before completing the task within the considered early window.

### Classification using XGBoost

To predict the outcome of (trimmed) clickstreams, we trained an extreme gradient boosting (XGBoost) classifier (Chen and Guestrin, 2016). Intuitively, XGBoost combines multiple weak learners—in the present application, classification trees—into a strong learner by aggregating the weak learners’ results. Our choice is motivated by XGBoost’s track record of exceptional performance in a variety of applications in general (Chen & Guestrin, 2016) and for the purpose of shopper intent prediction in particular (Requena et al., 2020). In addition, XGBoost has a built-in mechanism for dealing with missing values, which makes it particularly suitable for the problem at hand.

Each observation is represented by a *D*-dimensional feature vector $x \in \mathbb {R}^{D}$. A trained XGBoost classifier assigns to this observation a score $\hat {y} \in \mathbb {R}$, which, in turn, can be mapped to a binary class label.

#### Classification process

**Fig. 2:**
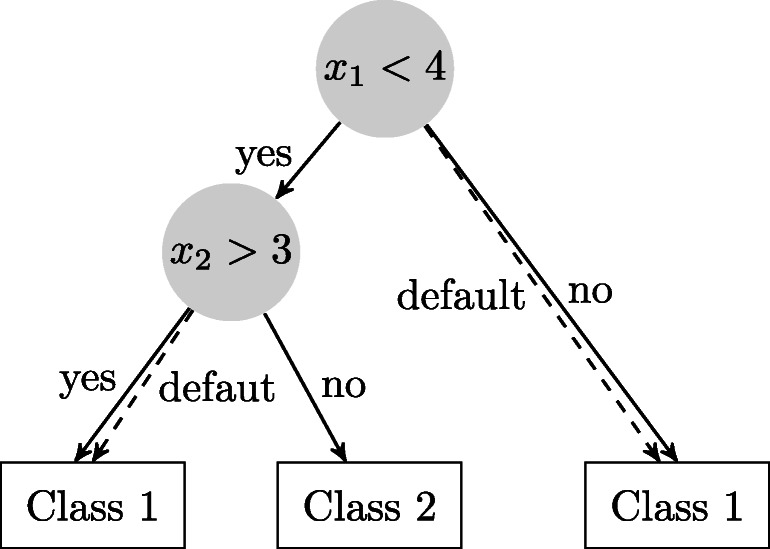
A depiction of a classification tree. An observation is classified by moving along the nodes of the tree depending on the values of the respective features. The dashed line indicates the default direction to take when the feature is missing. The leafs are associated with a score, which, in turn, is mapped to a class label

Before we turn our attention to training XGBoost classifiers, we first describe their classification process. An XGBoost classifier is composed of an ensemble of classification trees whose predictions are aggregated to reach a final decision. Figure 2 visualizes the classification process for a single tree. Classification trees are binary trees, where each node in the tree (except for the leaves) is associated with a feature $d \in \{1, \dots , D\}$ and a threshold value *δ*. To classify a given observation $x \in \mathbb {R}^{D}$, the observation traverses the binary tree, starting from the root, until it reaches a leaf node; the resulting leaf node will determine the classification. For traversing the tree, at each internal node, we move to the left child of the node if *x*_*d*_ ≥ *δ*, i.e., if the value of the *d* th feature of the observation is greater than the threshold value associated with the node, and to the right child otherwise. Each leaf is associated with a score $\hat {y}$. The sign of the score determines the class.

Once each classification tree mapped the observation to a leaf and the associated score, the XGBoost classifier aggregates the decision of the ensemble. Formally, the ensemble consists of *M* classification trees, modeled by a set of functions $\{f_{1}, \dots , f_{M}\}$, $f_{m}: \mathbb {R}^{D} \rightarrow \mathbb {R}, ~m \in \{1, \dots , M\}$. For a given observation $x \in \mathbb {R}^{D}$, XGBoost aggregates the decisions by taking the sum of the trees’ scores:

6 $$ \hat{y} = \sum\limits_{m=1}^{M} f_{m}(x). $$

To classify, the aggregated score $\hat {y}$ is then mapped to a class label.

#### Training

Given a training set of $N^{\text {train}}_{w}$ observations $X= \{x_{1}, \dots , x_{N^{\text {train}}_{w}}\}$, each associated with a binary class label *y*_*i*_ ∈{0,1}, XGBoost trains a tree ensemble sequentially. Intuitively, the training of the *m* th tree aims to remedy errors made by the previous *m*− 1 trees. This way, XGBoost iteratively improves (boosts) the predictor. The total number of trees *M* is a hyperparameter of XGBoost models. Each tree is trained to minimize an objective that corrects the residual errors of the previous predictors. Tree *m*,1 ≤ *m* ≤ *M*, is trained by minimizing the following objective:

7 $$ \sum\limits_{i=1}^{N^{\text{train}}_{w}} l(y_{i}, \hat{y}_{i}^{m-1} + f_{m}(x_{i})) + {\Omega}(f_{m}), $$

where $\hat {y}_{i}^{m-1} = {\sum }_{j=1}^{m-1} f_{j}(x_{i})$ denotes the aggregated decision of the first $m{}-{}1$ trained trees, *l*() is a loss-function, and Ω(*f*_*m*_) is a regularization term describing the complexity of the tree. In the present study, we use a logistic loss function. Intuitively, the complexity of a tree Ω(*f*_*m*_) is measured in terms of the magnitude of the scores assigned to the leaves and the depth of the tree.

To train a single tree, XGBoost adds nodes to the tree one after another, each time identifying the splitting point that maximizes improvement in the loss function. A splitting point is defined in terms of a feature and a threshold value. The algorithm iteratively transforms leafs into internal nodes until a predefined maximum depth is reached. For a more technical description we refer the reader to the standard literature (Chen & Guestrin, 2016).

One advantage of XGBoost is its built-in mechanism to deal with missing values. To this end, XGBoost implements so called sparsity-aware split finding. Each internal node in a tree is assigned a default direction (right or left) to take when the observation does not contain the feature that the respective node uses for splitting (see Fig. 2). During training, when identifying the best splitting point, XGBoost also chooses a corresponding default direction that results in a better training objective. In the present application, XGBoost’s ability to utilize informative missing values may aid in best leveraging the information contained in the times to first action occurrence. Recall that these not only contain information on when the respective actions were first performed but also on whether or not they were performed at all. In the latter case, times to first action were set to be missing.

### Set-up and implementation

For model selection and evaluation, we used nested cross validation. Nested cross validation has an outer loop with *k* folds for model evaluation and an inner loop that splits each of the *k* outer folds into *l* inner folds used for hyperparameter tuning. Following Requena et al., (2020), we used *l* = 3 inner folds and *k* = 5 outer folds.

#### Training data and hyperparameter optimization

To deal with imbalanced training data, we employed upsampling, i.e., replicating observations in the minority class to match the sample size of the majority class (Garcia, Sánchez, & Mollineda, 2012). In the inner cross validation loop, the optimal settings for the hyperparameters for each training sample of the outer cross validation loop were determined via grid search, varying the number of iterations (i.e., the number of trees to grow; 50; 100; 150), the maximum tree depth (3; 6; 9), and the learning rate (0.01; 0.10).^5^

All analyses were conducted in R version 3.6.3 (Core Team, 2020). We drew on the classification algorithm implemented in xgboost (Chen et al., 2021). The hyperparameter grid search of the inner cross-validation loop was performed using caret (Kuhn, 2021). Bi- and trigrams were extracted using ngram (Schmidt and Heckendorf, 2017). Exemplary R code is provided in the OSF repository accompanying this article.

### Evaluation criteria

**Fig. 3:**
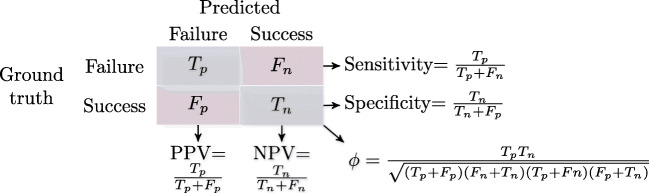
Confusion matrix and derived evaluation criteria

For evaluating predictions of failure, we monitored sensitivity, specificity, positive and negative predictive values (PPV and NPV), and coefficient *ϕ* (i.e., the correlation between observed and predicted failures) derived from the confusion matrix depicted in Fig. 3 alongside AUC ROC values.^6^ For constructing the confusion matrix, we set the discrimination threshold to .50, i.e., classified observations with probabilities of failure exceeding the threshold as failures. Note that failure was treated as the positive class (i.e., the class to be predicted).

### Feature evaluation

We leveraged XGBoost’s built-in feature importance calculation to get interpretable insights on the relevance of features for classification. For a given trained XGBoost model, the feature importance is calculated as the average gain across all splits the respective feature is used in (over all trees), where gain corresponds to the improvement of the loss function gained by splitting at a particular node.

## Results

### RQ1: Establishing a baseline

As evidenced in Table 3, for both items we achieved excellent classification performance when using complete clickstream data. This is not surprising, since clickstreams document examinees’ solution process, and, therefore, their pathways to success or failure, and merely serves as an indicator that XGBoost can well perform on the constructed features when sufficient information is available. While we could perfectly distinguish correct from incorrect sequences for “Lamp Return”, classification performance was slightly lower for “Meeting Rooms”.^7^

**Table 3:** Classification performance based on full clickstream data

Item	AUC	*ϕ*	Sensitivity	Specificity	PPV	NPV
U23 (“Lamp Return”)	1.00 (0.00)	1.00 (0.00)	1.00 (0.00)	1.00 (0.00)	1.00 (0.00)	1.00 (0.00)
U02 (“Meeting Rooms”)	0.98 (0.00)	0.86 (0.01)	0.91 (0.01)	0.96 (0.01)	0.97 (0.01)	0.88 (0.01)

Displayed are means and standard deviations across all five outer folds. AUC: Area under the receiver operating curve; PPV: positive predictive value; NPV: negative predictive value

For both items, classification of full sequences was almost exclusively performed based on information related to filling out the respective forms. On “Lamp Return”, Exchange_T, that is, the time elapsed until stating the reason for returning the lamp on the return form, was by far the most predictive feature, with a mean gain across all five outer folds of 0.94 and standard deviation of 0.02. On “Meeting Rooms”, Dept_tfidf, that is, tf-idf weights for selecting a department on the reservation form, was the most predictive feature, with a mean gain across all five outer folds of 0.75 and standard deviation of 0.01. For both items, all remaining features had mean gains below 0.05.

### RQ2: Investigating the accuracy of early predictions

**Fig. 4:**
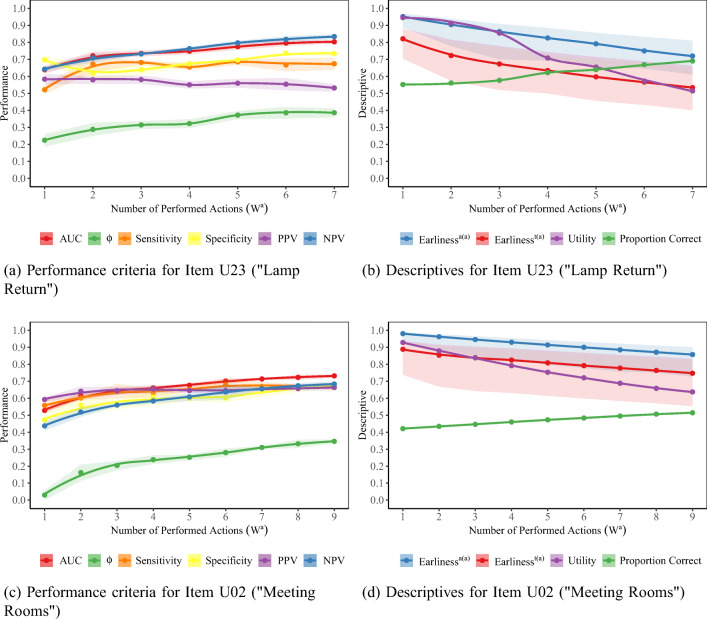
Evaluation of early-window predictability when trimming by the number of performed actions. Shaded areas for the performance criteria give the mean ± one standard deviation across the five outer folds. Solid lines for the Earliness metrics give their median, while shaded areas for the Earliness metrics denote middle 50% ranges within the respective early-window data set. AUC: Area under the receiver operating curve; PPV: positive predictive value; NPV: negative predictive value

**Fig. 5:**
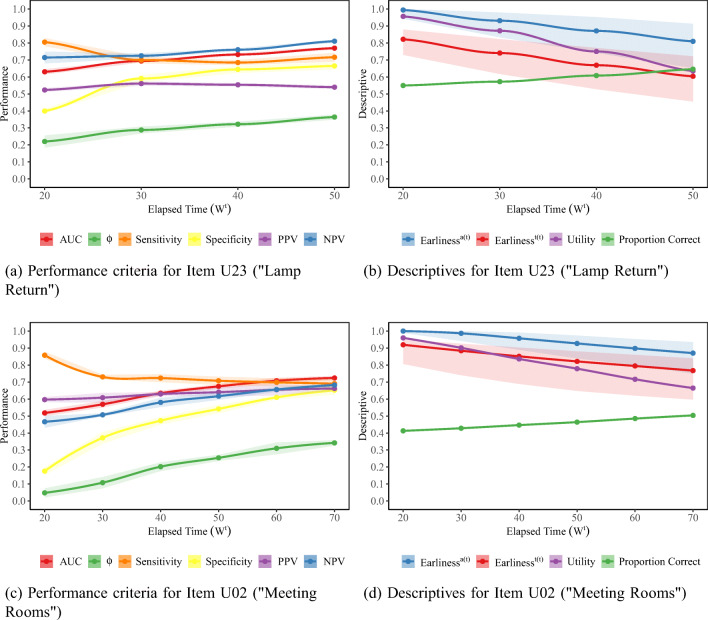
Evaluation of early-window predictability when trimming by elapsed time (in seconds). Shaded areas for the performance criteria give the mean ± one standard deviation across the five outer folds. Solid lines for the Earliness metrics give their median, while shaded areas for the Earliness metrics denote middle 50% ranges within the respective early-window data set. AUC: Area under the receiver operating curve; PPV: positive predictive value; NPV: negative predictive value

Figures 4 and 5 give classification performance values along with the monitored descriptives for the early-window data sets trimmed by actions and time, respectively. Note that for both tasks, Utility values (i.e., the ratio of the number of incorrect sequences in the early-window data set to the number of incorrect sequences in the complete data set) steadily declined from very early windows on. The initial drops in Utility go back to examinees leaving the task unsolved after performing no or only few actions in a short amount of time, presumably due to lack of motivation.

For early-window data sets created on the basis of the number of performed actions, classification performance in terms of all criteria rapidly improved with an increasing early-window size. Remarkably, for “Lamp Return”, information contained in the *w*^*a*^ = 1 data set was already sufficient to outperform classification at random chance level, as indicated by an AUC ROC value of 0.64. This was different for “Meeting Rooms”, where the AUC ROC value for *w*^*a*^ = 1 was 0.53. AUC ROC and *ϕ* values exceeded 0.70 and 0.30, respectively, at *w*^*a*^ = 3 on “Lamp Return” and at *w*^*a*^ = 7 on “Meeting Rooms”. At these points, comparable sensitivities (0.68 for “Lamp Return” and 0.67 for “Meeting Rooms”) and specificities (0.64 for both items) were achieved for both items; however, performance in terms of PPV and NPV differed. While for “Meeting Rooms”, PPV and NPV were comparable and acceptably high (0.66), “Lamp Return” yielded a relatively poor PPV of 0.58 combined with a high NPV of 0.73. These differences reflect the different base rates at the considered early-window sizes. While the *w*^*a*^ = 7 “Meeting Rooms” data set was balanced, the *w*^*a*^ = 3 “Lamp Return” data set contained a higher proportion of (partially) correct sequences (0.58).

The longer sequence length required to achieve classification performance for “Meeting Rooms” comparable to performance for “Lamp Return” with much shorter sequences reflects the higher complexity of “Meeting Rooms”. Note, however, that the raw action sequence lengths for which comparable classification performance was achieved correspond to comparable median Earliness^*a*(*a*)^ values (0.86 at *w*^*a*^ = 3 for “Lamp Return” and 0.88 at *w*^*a*^ = 7 for “Meeting Rooms”). That is, in relative terms, the items did not differ in their early predictability. Nevertheless, Utility at *w*^*a*^ = 7 for “Meeting Rooms” was lower (0.69) than at *w*^*a*^ = 3 for “Lamp Return” (0.86). That is, a lower proportion of examinees who failed could potentially have been identified to be at risk before completing the task.

By and large, we observed these patterns to mirror when early-window data sets were created on the basis of elapsed time (Fig. 5). The only exception were patterns for sensitivity and specificity. For both items, at *w*^*t*^ = 20 seconds, high sensitivities were accompanied by poor specificities. We could attribute these patterns to the fact that, due to lack of further information, all sequences not yet containing any actions after 20 s were classified as failures. On “Meeting Rooms”, 71% of examinees did not perform any actions within the first 20 s, out of which 60% of examinees failed. On “Lamp Return”, where the pattern was less extreme, 49% of examinees did not perform any actions within the first 20 s, and half of these examinees failed. With an increasing early-window size, more information for differentiating between these examinees became available—at *w*^*t*^ = 30 seconds, for instance, the proportion of sequences without any performed actions fell to 42% on “Meeting Rooms” and to 21% on “Lamp Return”—and sensitivities and specificities converged to each other. AUC ROC and *ϕ* exceeded 0.70 and 0.30 at *w*^*t*^ = 40 for “Lamp Return” and *w*^*t*^ = 60 for “Meeting Rooms”, corresponding to a median Earliness^*t*(*t*)^ of 0.69 and 0.79, respectively.

In additional analyses, we investigated variability of early predictability across examinees with different Earliness metrics for a given early window. As could be expected, we found that classification was more challenging for sequences for which the considered early window marked very early points of the solution process. Further details and full results are given in the Appendix.

### RQ3: Investigation of feature importance

**Fig. 6:**
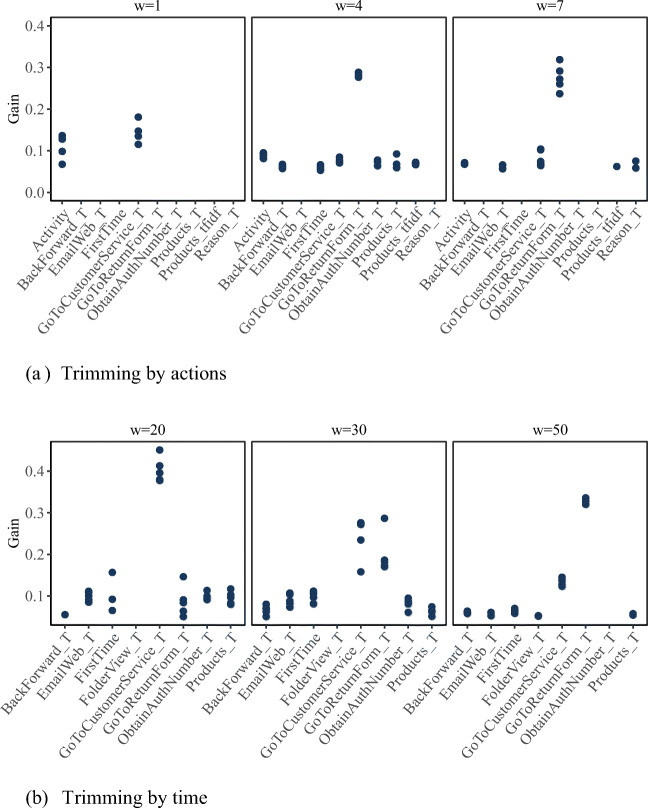
Feature importance for selected early windows for Item U23 (“Lamp Return”). Each dot refers to one of the outer validation folds. Only gains above 0.05 are displayed

**Fig. 7:**
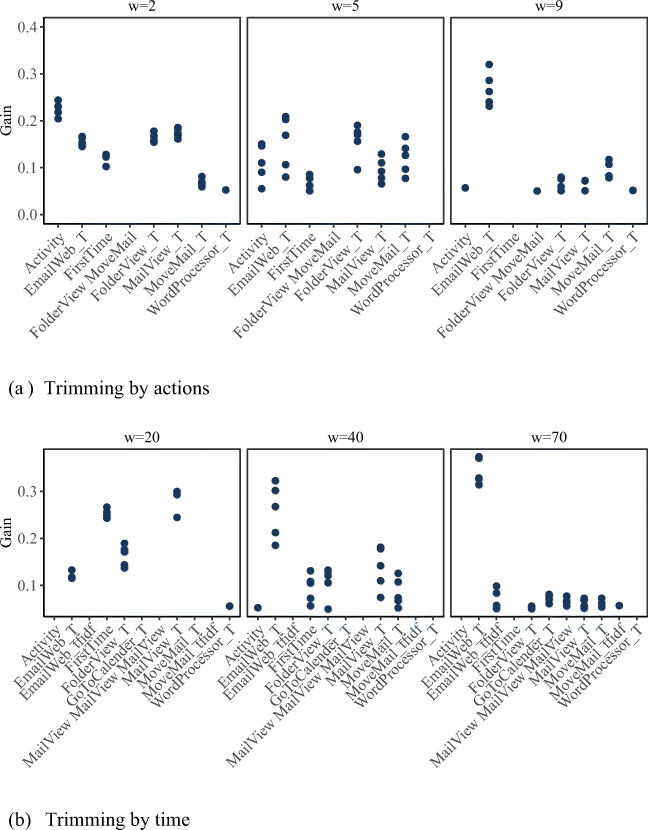
Feature importance for selected early windows for “Meeting Rooms”. Each dot refers to one of the outer validation folds. Only gains above 0.05 are displayed

Figures 6 and 7 exhibit changes in the importance of different features across selected early windows. For reasons of clarity, only those features are displayed for which gains above 0.05 were encountered in at least one of the outer validation folds. Notably, at very short early windows, features summarizing the intensity of examinees’ interactions with the task environment rather than referring to specific performed actions displayed the highest feature importance. This may indicate that these features reflect examinees’ reading and general computer competencies, determining how fast they can parse the task instructions and begin with their explorations as well as how fast they understand the task environment and are capable of navigating through it. With an increasing early-window size, features referring to specific actions gained increasing importance, documenting typical behavioral pathways.

For “Lamp Return”, in the *w*^*a*^ = 1 data set, by far the most important features were Activity (i.e., the time elapsed within the considered early window),^8^ and the time elapsed until first going to the online shop’s customer service site (see Fig. 6a). In the *w*^*a*^ = 4 and *w*^*a*^ = 7 data sets, the time elapsed until first visiting the online shop’s return form was the most important feature. Other important features were the time until first switching between sites and environments (BackForward_T and EmailWeb_T), the time to first exploring the online shops’ products (Products_T) as well as the intensity with which products were explored (Products_tfidf). Only in the *w*^*a*^ = 7 data set, the time until stating the reason for returning the lamp on the return form (Reason_T)—being performable only at later stages of the solution process—posed a relevant feature for classification. Similar patterns were observed when trimming by time (Fig. 6b). Here, the time elapsed until first going to the online shop’s customer service site was the most important feature in the *w*^*t*^ = 20 data set, while the time elapsed until first visiting the return form was the most important feature in the *w*^*t*^ = 50 data set. Note that examinees could only visit the return form via the customer service site, such that performing the former action was a prerequisite for the latter. The *w*^*t*^ = 30 data set can be seen as documenting a transition phase where both features showed comparable importance.

Analogously, for “Meeting Rooms”, Fig. 7 illustrates how the importance of the aggregate features Activity and FirstTime diminished with an increasing early-window size. For short early windows of *w*^*a*^ = 2 and *w*^*t*^ = 20, features related to exploring the task environment (FolderView_T and MailView_T) were of relevance. For *w*^*a*^ = 5 and *w*^*t*^ = 40, the time until examinees first moved an email (MoveMail_T) became relevant. Although this was not a necessary action for solving the task, the relevance of this feature may indicate that examinees moved emails containing completed reservation requests to other folders, thereby organizing themselves. A bigram and a trigram were among the most important features in the *w*^*a*^ = 9 and *w*^*t*^ = 70 data sets, highlighting the importance of taking the sequentiality of actions into account when sequences are longer. Interestingly, the time until first switching between the email and web environments of the task (EmailWeb_T) was a relevant feature throughout all considered early windows, and the intensity with which examinees did so (EmailWeb_tfidf) became a relevant feature in the *w*^*t*^ = 70 data set. This is not surprising, as examinees needed to compare and integrate information displayed in these environments, requiring (repeated) switching between them.

Recall that the time to first action occurrence was coded as missing when the respective action did not occur in a given sequence. Hence, the high importance of features related to the time of first action occurrence may be not only due to the time elapsed until examinees first performed the respective action, but also due to the informativeness of non-occurrence of the actions within the considered early windows. To support this hypothesis, however, further analyses are needed.

## Discussion

The aim of the present study was to introduce and showcase a machine learning-based procedure for systematically investigating early predictability of failure on interactive tasks based on early-window clickstream data. To that end, we analyzed two interactive tasks from the PIAAC 2012 PSTRE domain that differed in their difficulty and complexity of expert-defined solutions.

We enriched generic features derived from sequence data such as tf-idf encoded action occurrences and *n*-grams with information on time elapsed until the performance of actions. The employed XGBoost classifier trained on these features could almost perfectly distinguish failure from success when complete clickstreams were considered.

Building on procedures originating in shopper intent prediction, we created early-window data sets, stripping all time-stamped actions that occurred after a given number of actions or a given amount of time from the sequences, and investigated early predictability of failure on each of these data sets. This procedure supports a systematic investigation of how much information is needed to achieve sound early predictions. To evaluate the quality and utility of early predictions, we enriched customary machine learning performance metrics with Earliness and Utility measures.

We achieved AUC ROC values exceeding .70 and correlations between observed and predicted outcomes of above .30 at stages where examinees had, on average, at least more than two thirds of the number of actions to perform, respectively, time spent on task ahead of them, and the vast majority of examinees who failed could potentially be detected to be at risk before completing the task. This is remarkable, as the AUC ROC values achieved under these sparse data conditions resemble those reported for complete clickstreams using solely theory-derived features (Salles et al., 2020) or drawing on model-based rather than machine learning approaches for prediction at much later stages of the solution process (Chen et al., 2019). Nevertheless, we found predictability to differ across examinees for which the considered early windows marked different stages of the solution process.

In-depth analyses revealed different features to be indicative of success and failure at different stages of the solution process, thereby highlighting the potential of the applied procedure for gaining a finer-grained understanding of the trajectories of behavioral patterns on interactive tasks. Depending on how early predictions were performed, we found both aggregate features related to the timing and intensity of initially performed actions as well as the occurrence, timing, and frequency of single key actions of the solution process to be most important.

### Limitations and future research

Since the primary goals of the present study were to outline the procedure and provide a proof of concept for the utility of clickstream data for early prediction, we did not invest much effort into further improving our already fairly accurate prediction. As such, the reported performance criteria may be seen as a lower benchmark for future studies on further improving early predictability. Several directions can be taken to achieve this end. First, more elaborate features may be constructed, e.g., by incorporating expert knowledge into the construction of task-specific features. Second, indicators of examinees’ behavior on preceding tasks could be included. These could range from simple performance scores, indicating which tasks examinees were capable to solve correctly, through classifications into different behavioral groups (e.g., whether or not examinees employed some expert-defined strategy or showed behavior that may be classified as disengaged) to feature sets that provide a fine-grained summary of the preceding tasks’ clickstreams (e.g., as in Tang et al.,, 2020a). Third, future research may investigate whether other classifiers such as support vector machines or neural networks outperform XGBoost on features constructed from clickstream data. Fourth, the extraction of features may be left to deep learning approaches (see Urban & Gates, 2021, for an introduction), such as long short-term memory classifiers—a recurrent neural network architecture particularly well-suited for sequence data—as employed by Requena et al., (2020). Note, however, that such procedures sacrifice interpretability of predictive features and, therefore, do not support employing analyses of early-window predictive features for the purpose of better understanding examinees’ early solution behavior. Fifth, performance may be improved and customized by drawing on cost-sensitive learning (see Elkan, 2001). In cost-sensitive learning, each class is given a misclassification cost. For instance, researchers could make false predictions of success more costly than falsely identifying examinees as being at risk of failure. The classifier then aims to minimize the total misclassification cost instead of maximizing accuracy. Cost-sensitive learning can improve performance in imbalanced data (i.e., very easy or difficult tasks), when classifiers are “overwhelmed” by the majority class (Thai-Nghe, Gantner, & Schmidt-Thieme, 2010) and supports incorporating considerations on different costs of false positives and false negatives (Elkan, 2001).

For the tasks considered in the present study, different early-window sizes were required to achieve good predictability. The considered tasks differed in many aspects, such as difficulty, the number of performable actions, and the number and complexity of possible pathways to the correct solution, to name just a few. It remains an open task to investigate which of these aspects facilitate or hinder early predictability.

In showcasing the utility of clickstream data for early predictions in assessments, the procedure outlined in the present study may serve as a blueprint for studies investigating early predictability of other types of behavior and its outcomes and/or in other types of assessments with interactive modes. Collaborative, game- and simulation-based tasks, for instance, rapidly become more widely used (von Davier, Zhu, & Kyllonen, 2017) and commonly involve a myriad of behavioral outcomes besides success or failure, such as motivational or affective outcomes (e.g., flow experience or reduced test anxiety) in game-based assessments (Kiili and Ketamo, 2017) or the effectiveness of cooperation in collaborative problem-solving (Hao, Liu, von Davier, Kyllonen, & Kitchen, 2016). In low-stakes assessments, the predictability of disengaged test-taking behavior may be particularly worth investigating. On the basis of such predictions, attempts can be taken to motivate examinees identified to be at risk of showing disengaged test-taking behavior to display the best of their abilities, thereby increasing validity of conclusions drawn from low-stakes assessment data (see Wise, Bhola, & Yang, 2006, for an application on multiple choice items).

From a methodological perspective, the present study once again highlights the vast potential of employing methods for real-life clickstream data (i.e., user’s interactions with websites) for understanding clickstream data collected on interactive tasks (see He et al.,, 2021; Tang et al.,, 2020a; Ulitzsch et al.,, 2021b, for applications in psychometrics). While the analysis of such data has only recently gained the attention of the psychometric, psychological, and educational science communities, clickstream analyses of users’ interactions with websites has a much longer history, offering a variety of methods that are worth to further explore and adapt to the context of educational assessment.

## Conclusion and implications

Using machine learning techniques on early-window clickstream data supports making sound early predictions of failure or success on interactive tasks as well as a finer-grained understanding of examinees’ initial exploration and solution behavior. Insights gained from such analyses can be of utility for both improving assessments and deriving appropriate conclusions and interventions from their results. First, understanding what distinguishes initial task exploration and solution behavior leading to success or failure may aid in improving the construction of interactive tasks. Item developers can closely investigate whether examinees explore the task environment as intended or whether there are some elements that are confusing or misleading and lead to failure.

Second, early identification of examinees at the risk of failure provides the opportunity to support these examinees in their solution process, e.g. by providing hints or further clarifications, and systematic investigations of early predictability may aid in identifying the optimal point to provide such support. The adaptive tailoring of the tasks’ difficulties to examinees’ skill levels could then mirror the principles of computer-adaptive testing, possibly resulting in a more engaging assessment experience for examinees and more precise proficiency estimates. As such, the present study could also be understood as a pilot study to initiate discussions on new forms of adaptive testing, where, instead of the items to be administered, the tasks themselves are subject to adaptations based on real-time evaluations of examinees’ competencies. It should be noted, however, that before such adaptive items can be developed, different types of failure need to be distinguished and separately identified in the early prediction model. Failure on interactive tasks can occur for a variety of different reasons, ranging from lack of different subskills and/or metacompetencies required to solve the task through misinterpreting instructions to examinees not exerting their best effort and interacting quickly and superficially with the task at hand (Ulitzsch et al., 2021a), and different types of predicted failure may require different types of item adaptations.

Third, understanding how low proficiency examinees initially explore the task environment may aid in designing interventions aimed at improving problem-solving skills by equipping examinees with strategies on how to more effectively explore and approach problem-solving tasks, following recent calls to better target interventions at specific subskills or metacompetencies examinees are lacking (Stadler, Fischer, & Greiff, 2019).

## Variability of Early Predictability for Examinees With Different Earliness Metrics

To investigate variability of early predictability across examinees with different Earliness metrics for a given early window, we evaluated performance measures separately for each Earliness tertile for some selected early windows. We exemplarily focused on those early windows for which AUC ROC and *ϕ* first exceeded 0.70 and 0.30, respectively. That is, we considered *w*^*a*^ = 3 and *w*^*t*^ = 50 for “Lamp Return” and *w*^*a*^ = 7 and *w*^*t*^ = 70 for “Meeting Rooms”. We expected the quality of early prediction to vary across Earliness tertile groups. First, for examinees with higher Earliness parameters, prediction is performed at earlier stages of the solution process and key actions differentiating success from failure may not yet have been performed. Second, the different sequence lengths, respectively times spent on task, reflected in differences in Earliness for a given early window may be indicative of different subpopulations of behavioral patterns leading to success or failure on the task. Short time spent on task and few performed actions may stem from disengaged test-taking behavior when associated with failure (Sahin & Colvin, 2020; Ulitzsch et al., 2021a), but may be indicative of high competency and an efficient response strategy when associated with success (Hao et al., 2015). A high number of performed actions, respectively long time spent on the task, may go back to examinees trying hard to solve the item, but having an inefficient strategy or showing extensive, unsystematic, or idiosyncratic exploration behavior (Ulitzsch et al.,, 2021a, 2021b). Early-window behavioral patterns distinguishing success from failure in these different subpopulations and, as a consequence, predictability may differ. Note that we employed the prediction models trained on the whole early-window data set and did not train separate models for each tertile group, following the consideration that when dynamically predicting failure under real-life assessment conditions, Earliness for a given early window is unknown until examinees complete the task.

Results are displayed in Table 4. As expected, we observed differences in classification performance across the Earliness tertile groups. Across all considered early-window data sets, AUC ROC values decreased across the Earliness tertile groups. That is, classification was more challenging for sequences for which the considered early window marked very early points of the solution process. The same is true for specificities as well as for sensitivities and coefficient *ϕ* on “Lamp Return”. The extreme imbalancedness in “Meeting Rooms”’s lower and upper tertile groups seemed to challenge coefficient *ϕ*, resulting in an inverted U-shape across the Earliness tertile groups. Interestingly, sensitivities on “Meeting Rooms” decreased in the *w*^*a*^ data set, but were U-shaped in the *w*^*t*^ data set. Strong PPV decreases accompanied by NPV increases across the Earliness tertile groups partially reflect the different base rates (i.e. proportions correct) within the groups. This becomes particularly evident for the lower tertile groups for “Meeting Rooms”, where sequence lengths were too short to lead to (partial) success and, hence, almost all sequences were associated with failure. Given the extremely low proportions correct, the PPV close to 1 and NPV close to 0 are not surprising.

**Table 4:** Earliness tertile group-specific classification performance for selected early window data sets

Early window	Tertile	Earliness	Proportion correct	AUC	*ϕ*	Sensitivity	Specificity	PPV	NPV
Item U23 (“Lamp Return”)									
*w*^*a*^ = 3	1	0.57	0.11	0.91 (0.03)	0.37 (0.05)	0.70 (0.02)	0.87 (0.05)	0.98 (0.01)	0.27 (0.03)
	2	0.86	0.80	0.75 (0.03)	0.28 (0.05)	0.69 (0.05)	0.66 (0.04)	0.33 (0.04)	0.90 (0.01)
	3	0.92	0.80	0.63 (0.03)	0.14 (0.04)	0.58 (0.05)	0.59 (0.03)	0.26 (0.04)	0.85 (0.01)
*w*^*t*^ = 50	1	0.38	0.48	0.94 (0.01)	0.64 (0.04)	0.68 (0.02)	0.94 (0.02)	0.93 (0.03)	0.73 (0.02)
	2	0.60	0.73	0.80 (0.02)	0.39 (0.03)	0.74 (0.06)	0.69 (0.02)	0.47 (0.03)	0.87 (0.03)
	3	0.76	0.73	0.66 (0.02)	0.20 (0.03)	0.76 (0.07)	0.46 (0.05)	0.35 (0.02)	0.84 (0.03)
Item U02 (“Meeting Rooms”)									
*w*^*a*^ = 7	1	0.63	0.04	0.78 (0.07)	0.17 (0.07)	0.71 (0.03)	0.68 (0.17)	0.98 (0.01)	0.09 (0.03)
	2	0.88	0.65	0.72 (0.03)	0.34 (0.03)	0.62 (0.03)	0.73 (0.02)	0.55 (0.03)	0.78 (0.01)
	3	0.93	0.78	0.61 (0.04)	0.12 (0.04)	0.58 (0.05)	0.57 (0.04)	0.27 (0.02)	0.83 (0.02)
*w*^*t*^ = 70	1	0.47	0.07	0.90 (0.02)	0.36 (0.04)	0.72 (0.03)	0.94 (0.03)	0.99 (0.00)	0.20 (0.03)
	2	0.77	0.63	0.76 (0.03)	0.42 (0.07)	0.59 (0.07)	0.81 (0.04)	0.65 (0.06)	0.77 (0.02)
	3	0.86	0.80	0.66 (0.04)	0.20 (0.05)	0.74 (0.06)	0.51 (0.04)	0.27 (0.02)	0.89 (0.03)

Displayed are means and standard deviations across all five outer folds. AUC: Area under the receiver operating curve; PPV: positive predictive value; NPV: negative predictive value; Earliness gives mean Earliness^*a*(*a*)^ within the respective tertile group when trimming by actions and mean Earliness^*t*(*t*)^ within the respective tertile group when trimming by time

## Footnotes

1 Note that the word processor is an optional environment instead of a compulsory one, designed to assist examinees to summarize information extracted from the email requests.

2 The PSTRE performance is defined by four levels: below Level 1 (0–240), Level 1 (241–290), Level 2 (291–340) and Level 3 (341–500). For more details, refer to OECD (2013).

3 The manifold ways of how this initial task exploration manifests itself in action sequences is, among others, reflected in the comparably low similarity of sequences to expert-defined optimal strategies (He et al., 2019).

4 We did not consider early windows of 10 s, as on “Lamp Return” only 6% and on “Meeting Rooms” only 4% of examinees performed their first action within the first 10 s. Within the first 20 s—the shortest time-wise early window considered—51% and 29% of examinees already performed their first action on “Lamp Return” and “Meeting Rooms”, respectively.

5 Intuitively, the learning rate refers to the weight given to the new trees’ corrections of the residual errors when added to the model. The learning rate can take values between 0 and 1.

6 Recall that the ROC curve is plotted with the true positive rate (i.e., sensitivity) on the y-axis against the false positive rate (i.e., 1 −specificity), on the x-axis under varying discrimination thresholds. Hence, the AUC ROC poses a combined measure of sensitivity and specificity and equals 0.5 when the ROC curve corresponds to random chance and 1.0 for perfect accuracy.

7 These results reflect the higher complexity of the task. While almost all examinees who opened the return form were able to fill it out (partially) correctly on “Lamp Return” (only 3% of sequences associated with failure contained interactions with the return form, see Table 1), 27% of examiness who failed on “Meeting Rooms” opened the reservation form but did not provide correct information (see Table 2). That is, information on whether examinees interacted with the form is sufficient for classification on “Lamp Return”, but not on “Meeting Rooms”, where additional information on the correctness of information provided on the reservation form is needed to achieve perfect classification. Recall that we stripped this information from the sequences.

8 Note that for *w*^*a*^ = 1, Activity corresponds to the time to first action, since only one action was performed within the early window.
